# Cariprazine in Obsessive–Compulsive Disorder: A Three-Case Series

**DOI:** 10.1192/j.eurpsy.2026.11602

**Published:** 2026-07-31

**Authors:** A. Karampas, D. D. Koutsogianni, A. P. Taprantzi, A. Goudeli, A. Kοtsi, C. Pappa, P. Petrikis

**Affiliations:** Psychiatry, Faculty of Medicine, University of Ioannina, Ioannina, Greece

## Abstract

**Introduction:**

Obsessive–Compulsive Disorder (OCD) is a chronic and often treatment-resistant condition that significantly affects functioning and quality of life. OCD affects an estimated 1–2% of the general population in Europe. Patients with OCD experience persistent, unwanted thoughts (obsessions) and/or repetitive behaviors or rituals (compulsions), which frequently cause significant distress and impairment in daily life. . Despite the efficacy of serotonin reuptake inhibitors (SRIs), a group of patients show only partial response, requiring augmentation strategies. Cariprazine, a dopamine D3/D2 receptor partial agonist with preferential D3 affinity, has been shown to improve motivational and cognitive symptoms in schizophrenia and depression. Its potential role in OCD treatment remains under investigation.

**Objectives:**

Presentation of three clinical cases of patients with OCD who were treated with cariprazine as an augmentation strategy to SSRIs.

**Methods:**

**Case Descriptions:**

- one female 24 years old with OCD diagnosis, previous treatment with Sertraline, Quetiapine and current treatment with Sertraline 200mg Cariprazine 3 mg for 4 months and an outcome of improvement in depressive and anxiety symptoms, reduction in frequency and intensity of obsessions.
- one male 50 years old with OCD diagnosis, previous treatment with Sertraline, Fluoxetine, Paroxetine, Aripiprazole and current treatment Paroxetine 30mg, Cariprazine 3mg for 5 months and an outcome of reduction of anxiety symptoms and obsessions and mild sexual dysfunction.
- one male 36 geras old with OCD, previous treatment with Escitalopram, Fluoxetine, Olanzapine, Aripiprazole and current treatment with Fluoxetine 60mg, Cariprazine 4,5mg for 12 months and an outcome of marked reduction of stress and health related obsessions.

**Results:**

All three patients showed clinically meaningful improvement in obsessive–compulsive and anxiety symptoms, as well as overall functioning. A statistically significant reduction in Y-BOCS scores was observed in all three cases after the initiation of cariprazine treatment. Cariprazine was well tolerated: only one patient reported mild sexual dysfunction. No extrapyramidal or metabolic adverse effects were observed.

**Discussion:**

These cases suggest that cariprazine may be an effective augmentation option for patients with OCD who show inadequate response to SSRIs or other augmentation strategies. The improvement in cognitive symptoms observed in these cases may relate to cariprazine’s D3-preferring pharmacodynamic profile.

**Conclusions:**

Cariprazine appears to be a promising adjunctive treatment for OCD, particularly in patients with residual anxiety, depressive, or motivational symptoms. Its favorable tolerability profile and potential benefits on cognitive and affective domains make it a candidate for further research.

**Disclosure of Interest:**

None Declared

